# Cerebral Blood Flow Changes in Multiple Sclerosis and Neuromyelitis Optica and Their Correlations With Clinical Disability

**DOI:** 10.3389/fneur.2018.00305

**Published:** 2018-05-02

**Authors:** Xue Zhang, Xi Guo, Ningnannan Zhang, Huanhuan Cai, Jie Sun, Qiuhui Wang, Yuan Qi, Linjie Zhang, Li Yang, Fu-Dong Shi, Chunshui Yu

**Affiliations:** ^1^Department of Radiology, Tianjin Key Laboratory of Functional Imaging, Tianjin, China; ^2^Laboratory of Digital Medical Imaging, Medical Imaging Center, The First Affiliated Hospital, Anhui University of Chinese Medicine, Hefei, China; ^3^Department of Neurology, Tianjin Medical University General Hospital, Tianjin, China

**Keywords:** arterial spin labeling, relapsing-remitting multiple sclerosis, neuromyelitis optica, cerebral blood flow, perfusion

## Abstract

Distinguishing relapsing-remitting multiple sclerosis (RRMS) and neuromyelitis optica (NMO) is clinically important because they differ in prognosis and treatment. This study aimed to identify perfusion abnormalities in RRMS and NMO and their correlations with gray matter volume (GMV) atrophy and clinical parameters. Structural and arterial spin labeling MRI scans were performed in 39 RRMS patients, 62 NMO patients, and 73 healthy controls. The gray matter cerebral blood flow (CBF) values were voxel-wisely compared among the three groups with and without GMV correction. The regional CBF changes were correlated with the Expanded Disability Status Scale scores in the corresponding patient groups. Although multiple brain regions showed CBF differences among the three groups without GMV correction, only three of these regions remained significant after GMV correction. Specifically, both the RRMS and NMO groups showed reduced CBF in the occipital cortex and increased CBF in the right putamen compared to the control group. The RRMS group had increased CBF only in the medial prefrontal cortex compared to the other two groups. The occipital CBF was negatively correlated with clinical disability in the NMO group; however, the CBF in the right putamen was positively correlated with clinical disability in both patient groups. These findings suggest that there are perfusion alterations independent of GMV atrophy in RRMS and NMO patients. The regional CBF in the occipital cortex and putamen could be used as imaging features to objectively assess clinical disability in these patients.

## Introduction

As the most common demyelinating diseases of the central nervous system in Asian populations (1, 2), multiple sclerosis (MS) and neuromyelitis optica (NMO) show both common and distinct features in terms of symptoms and immunological, pathological, and imaging aspects (3–5). Identification of the similarities and differences in these features between the two disorders would be very helpful in understanding the pathogenic mechanisms and in making differential diagnoses. Over the past few decades, brain MRI techniques have greatly improved our understanding of the brain damage in these two disorders. In addition to the extensive white matter damage, both disorders have shown the involvement of gray matter (6–16). However, the absence of cortical lesions and atrophy or deep gray matter abnormalities has also been reported in NMO or NMO spectrum disorder (NMOSD) with aquaporin-4 antibody-positive (AQP4 Ab-positive) (17–20).

Despite the lack of reliable evidence for the cortical atrophy in NMO, gray matter volume (GMV) reductions were found in both MS and NMO in a previous work (10). The reduction in cell components theoretically reduces both the metabolic and energy consumption and then results in cerebral blood flow (CBF) changes in these disorders. Moreover, the impairments in neurons, astrocytes, and microvascular components (21, 22) due to disease pathology may lead to neurovascular decoupling, which also resulting in CBF changes in these disorders. More importantly, the gray matter damage in these diseases is critically important because it can substantially affect clinical cognitive function (9, 23, 24). As expected, many studies using different imaging methods have revealed gray matter CBF changes in patients with MS (3, 6–8, 10–12, 15). However, only one study has investigated and found gray matter CBF changes in patients with NMO (13).

Because MS and NMO differ in terms of both prognosis and approaches to treatment (25–29), it is clinically important to distinguish MS from NMO. Although immunological, clinical, and imaging measures have exhibited the potential to distinguish these two disorders, no method has been able to completely differentiate MS from NMO. Thus, there is an urgent need to identify imaging markers that could be used to distinguish these two disorders. Currently, none of the existing studies are performed to compare the resting-state CBF differences in gray matter between MS and NMO. Investigation of CBF changes in MS and NMO may help not only to identify potential imaging measures for distinguishing these two diseases but also to understand the pathogenic mechanisms of these disorders.

Because GMV atrophy has been shown to affect CBF changes in the brain, we aimed to identify CBF differences among the relapsing-remitting MS (RRMS), NMO, and control groups that are independent of GMV reductions. The clinical significance of the potential CBF changes was assessed by investigating their correlations with the clinical disabilities in these disorders.

## Materials and Methods

### Subjects

This study was approved by the Medical Research Ethics Committee at Tianjin Medical University General Hospital, and the participants provided informed written consent. By using a database for patients with NMO or MS who had visited Tianjin Medical University General Hospital for treatment within the prior 5 years, all patients were invited to participate in this experiment during the recruitment phase. The healthy controls (HC) were recruited *via* advertisements in the local community. A total of 39 RRMS patients, 62 NMO patients, and 73 HC were included in this study (Table 1). The inclusion criteria for both the patients and controls were an age of 18–70 years and right-handedness. The RRMS patients fulfilled the revised McDonald criteria (30), and the NMO patients satisfied the revised Wingerchuk diagnostic criteria (31) (Table S1 in Supplementary Material). Moreover, a retrospective evaluation confirmed that they also fulfilled the 2015 criteria for NMOSD (32) (Table S2 in Supplementary Material). The AQP4 antibody was tested using a cell-based array through quantitative flow cytometry; there were 40 NMO patients who were seropositive for AQP4 antibodies (details and results relating to the assay protocol were shown in Supplementary Material). The prevalence of optic neuritis and myelitis among the MS patients were 24 and 81%, respectively, while both were 100% among the NMO patients.

**Table 1:** Demographic and clinical characteristics of the participants.

Participants	RRMS patients (*n* = 39)	NMO patients (*n* = 62)	Healthy controls (*n* = 73)	*p* Value
Sex (F/M)	23/16	53/9	55/18	0.011
Age (years)	38.72 ± 12.64	47.68 ± 13.91	48.14 ± 9.83	<0.001
EDSS scores	2 (0–6)	3.5 (0–9)	–	<0.001
Disease duration	4.22 ± 4.94	6.12 ± 6.48	–	0.144

*EDSS scores reported as median (range), the rest values are expressed as mean ± SD*.

*EDSS, Expanded Disability Status Scale; RRMS, relapsing-remitting multiple sclerosis; NMO, neuromyelitis optica*.

All MS and NMO patients exhibited the relapsing form and were recruited during the chronic stage, having not taken corticosteroids within 1 month. The exclusion criteria for both the patients and controls included MRI contraindications; histories of head trauma, neuropsychiatric diseases or other autoimmune diseases; and poor image quality (visible artifacts). The disease severity of the patients was assessed using the Expanded Disability Status Scale (EDSS) scores.

### MRI Data Acquisition

MRI data were acquired using a 3.0-T MR system (Discovery MR750, General Electric, Milwaukee, WI, USA). Tight but comfortable foam padding was used to minimize head motion, and earplugs were used to reduce noise. Conventional brain and spinal cord MRI scans were performed to detect visible lesions. Sagittal 3D T1-weighted images were acquired using a brain volume sequence with the following parameters: repetition time (TR) = 8.2 ms; echo time (TE) = 3.2 ms; inversion time (TI) = 450 ms; flip angle (FA) = 12°; field of view (FOV) = 256 mm × 256 mm; matrix = 256 × 256; slice thickness = 1 mm, no gap; and 188 sagittal slices. The resting-state perfusion imaging was performed using a pseudo-continuous ASL (pcASL) sequence with a 3D fast spin-echo acquisition and background suppression (TR/TE = 4,886/10.5 ms; post-label delay = 2,025 ms; spiral in readout of eight arms with 512 sample points; FA = 111°; FOV = 240 mm × 240 mm; reconstruction matrix = 128 × 128; slice thickness = 4 mm, no gap; 40 axial slices; number of excitations = 3; and acquisition time = 284 s). The label and control whole-brain volumes required eight TRs, respectively. A total of three pairs of label and control volumes were acquired. During the ASL scans, all subjects were instructed to keep their eyes closed, to relax and move as little as possible, to think of nothing in particular, and not to fall asleep.

### CBF Calculation

The ASL difference images were calculated through the subtraction of the label images from the control images. The CBF maps were subsequently derived from the ASL difference images. The detailed calculation procedures were described in a previous study (33, 34). The CBF images of all participants were coregistered to the standard CBF template in the Montreal Neurological Institute (MNI) space and were resampled to 2-mm cubic voxels using the Statistical Parametric Mapping (SPM8^1^). Each coregistered CBF map was removed of non-brain tissue and spatially smoothed with a Gaussian kernel of 8 mm × 8 mm × 8 mm full-width at half maximum (FWHM). For each participant, the CBF maps were further normalized into *z* scores by subtracting the mean and dividing by the SD of the global value within the gray matter mask. As a result, 0 represents the mean of the global CBF values, while the positive and negative values represent higher and lower than the mean, respectively.

### GMV Calculation

The GMV of each voxel was calculated using the SPM8 software. The structural MR images were segmented into gray matter, white matter, and cerebrospinal fluid using the standard unified segmentation model. After an initial affine registration of the gray matter concentration map to the MNI space, the maps were nonlinearly warped using the diffeomorphic anatomical registration through the exponentiated Lie algebra (DARTEL) technique (35) and were resampled to a voxel size of 2.0 mm × 2.0 mm × 2.0 mm. The GMV of each voxel was obtained by multiplying the GM concentration map by the non-linear determinants derived from the spatial normalization step. Finally, the GMV images were smoothed with a Gaussian kernel of 8 mm × 8 mm × 8 mm FWHM. After spatial preprocessing, the normalized, modulated, and smoothed GMV maps were used for the statistical analyses.

### Statistical Analysis

The demographic and clinical data were analyzed using the Statistical Package for the Social Sciences version 19.0 (SPSS, Chicago, IL, USA). The chi-square test was used to compare the gender differences among the three groups, the non-parametric Kruskal–Wallis ANCOVA test was used to evaluate the age differences among the three groups, and the non-parametric Mann–Whitney *U* test was used to assess the differences in the clinical data between the two patient groups.

Non-parametric tests were performed to investigate voxel-wise GMV and CBF differences among the RRMS, NMO, and HC groups, while controlling for the effects of age and gender. To exclude the potential confounding effect of GMV atrophy on the CBF comparisons, we also repeated the voxel-based CBF analyses with the GMV of each voxel as an additional covariate of no interest. Multiple comparisons for these voxel-wise analyses were corrected using permutation-based non-parametric testing (Randomize v2.1 of FSL^2^). The number of permutations was 5,000, and the significance threshold for intergroup differences was set at *P* < 0.05 after correcting for family-wise error (FWE) using the threshold-free cluster enhancement option in the permutation-testing tool in FSL. For each subject, the normalized CBF of each cluster with a significant group difference was extracted and used for region of interest-based analyses. With age and sex as covariates of no interest, the Kruskal–Wallis ANCOVA test was used to test CBF differences among the three groups and then *post hoc* comparisons were used to identify CBF differences between every two groups (*P* < 0.05). For clusters with significant CBF changes in the RRMS or NMO group, we performed Spearman correlations between the CBF and GMV values in each patient group to explore their relationships. In addition, Spearman correlations were also performed between the CBF values of these clusters and EDSS scores. In addition, voxel-wise correlation analyses between the gray matter CBF values and EDSS scores were also performed in each patient group.

## Results

### Demographic and Clinical Characteristic of Participants

The demographic and clinical data of the RRMS patients, NMO patients, and HC are shown in Table 1. There were significant differences in terms of both sex (*P* = 0.011) and age (*P* < 0.001) among the three groups. Specifically, the NMO patients showed a significant female predominance compared to the RRMS patients (*P* = 0.003); the RRMS patients demonstrated relatively younger ages than both the NMO patients (*P* = 0.001) and HC (*P* = 0.001); and the NMO patients had greater EDSS scores (*P* < 0.001) than the RRMS patients.

### The CBF Differences Among the Three Groups Without GMV Correction

**Figure 1:**
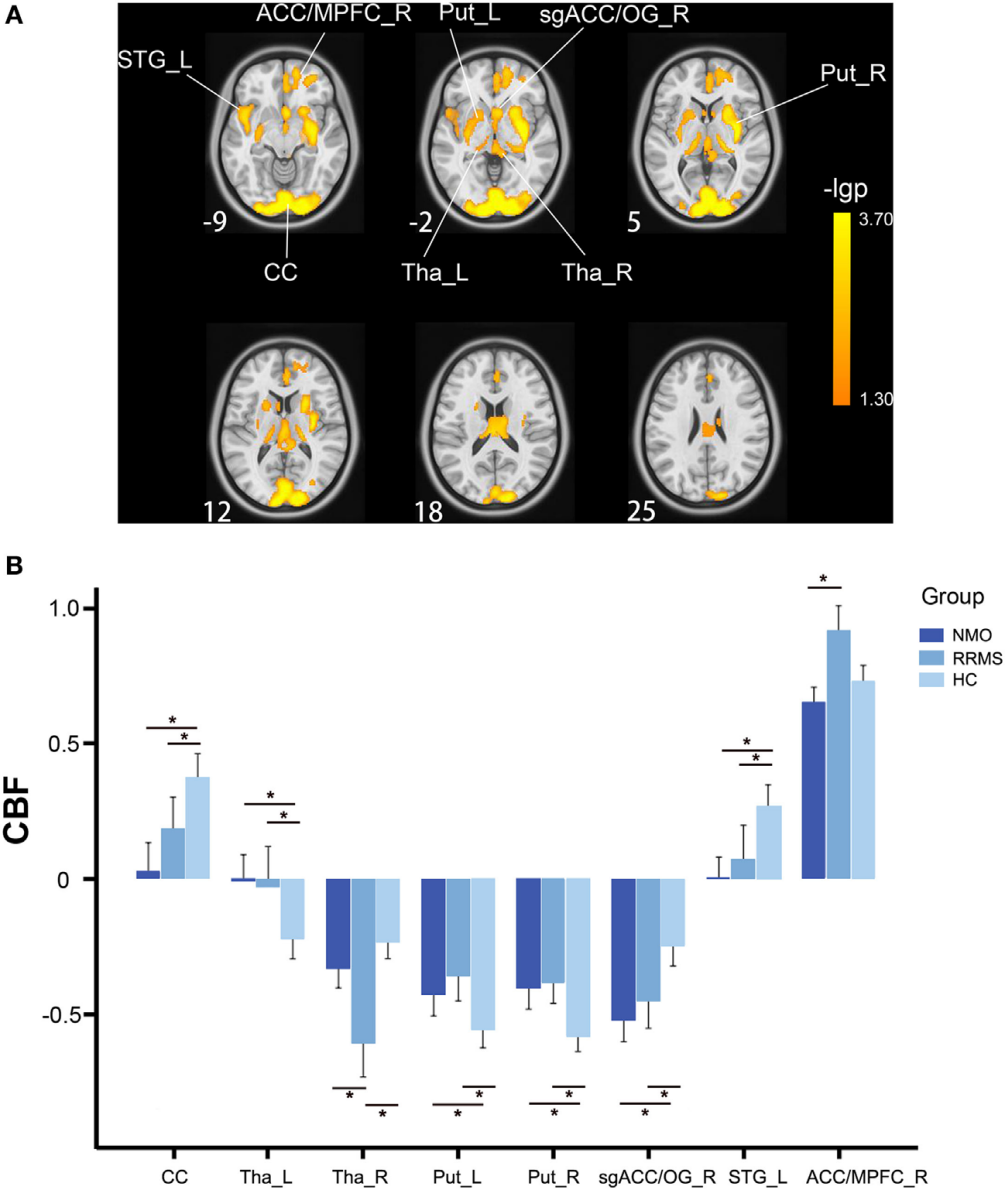
CBF differences among the relapsing-remitting multiple sclerosis, NMO, and control groups without GMV correction (*P* < 0.05, FWE-corrected). **(A)** Voxel-based analysis shows brain regions with significant CBF differences among the three groups using age and gender as nuisance variables (*P* < 0.05, FWE-corrected). **(B)** Bar plots show CBF differences between every two groups. Error bars are SEs. *P < 0.05. Abbreviations: ACC, anterior cingulate cortex; CBF, cerebral blood flow; CC, calcarine cortex; FWE, family-wise error; L, left; MPFC, medial prefrontal cortex; Put, putamen; R, right; sgACC, subgenual anterior cingulate cortex; STG, superior temporal gyrus; Tha, thalamus.

After controlling for the effects of age and gender, the bilateral calcarine cortex (CC), thalamus and putamen, the left superior temporal gyrus (STG), the right subgenual anterior cingulate cortex (ACC) and olfactory gyrus [subgenual anterior cingulate cortex (sgACC)/OG], and the right ACC and medial prefrontal cortex (ACC/MPFC) showed significant CBF differences (*P* < 0.05, FWE-corrected) among the three groups (Figure 1A; Table 2).

**Table 2:** Brain regions with significant group differences in CBF.

Regions	Brodmann areas	Cluster size (voxels)	Peak *p* values	Coordinates in MNI (*x*,*y*,*z*)
**Without GMV correction**				
CC	17, 18	4,839	0.0002	−14, −98, −16
Tha_L	–	202	0.0048	−16, −24, 4
Tha_R	–	1,049	0.0016	−2, −26, 16
Put_L	–	617	0.0048	−30, −14, −4
Put_R	–	1,884	0.0002	32, −18, −8
sgACC/OG_R	25	493	0.0008	2, 12, −14
STG_L	38,13	507	0.0012	−44, 8, −12
ACC/MPFC_R	11,32,10	1,648	0.0024	14, 56, −10

**After GMV correction**				
CC	18	4,444	0.0002	−14, −98, −16
Put_R	–	1,458	0.0004	32, −20, −6
ACC/MPFC_R	11,32,10	962	0.0052	−14, 58, −6

*ACC, anterior cingulate cortex; CBF, cerebral blood flow; CC, calcarine cortex; GMV, gray matter volume; L, left; MNI, Montreal Neurological Institute; MPFC, medial prefrontal cortex; Put, putamen; R, right; sgACC, subgenual anterior cingulate cortex; STG, superior temporal gyrus; Tha, thalamus*.

The *post hoc* analyses revealed that both the RRMS and NMO patients showed increased CBF in the left thalamus (*P* < 0.001 for both RRMS and NMO) and the bilateral putamen (left: *P* < 0.001 for RRMS and *P* = 0.002 for NMO; right: *P* < 0.001 for both RRMS and NMO), and decreased CBF in the bilateral CC (*P* = 0.018 for RRMS and *P* < 0.001 for NMO), the right sgACC/OG (*P* < 0.001 for both RRMS and NMO), and the left STG (*P* < 0.001 for both RRMS and NMO) compared to the HC. The RRMS patients had reduced CBF in the right thalamus compared to the NMO patients (*P* = 0.013) and HC (*P* < 0.001). Moreover, the RRMS patients showed increased CBF in the right ACC/MPFC compared to the NMO patients (*P* < 0.001) (Figure 1B).

### The CBF Differences Among the Three Groups With GMV Correction

**Figure 2:**
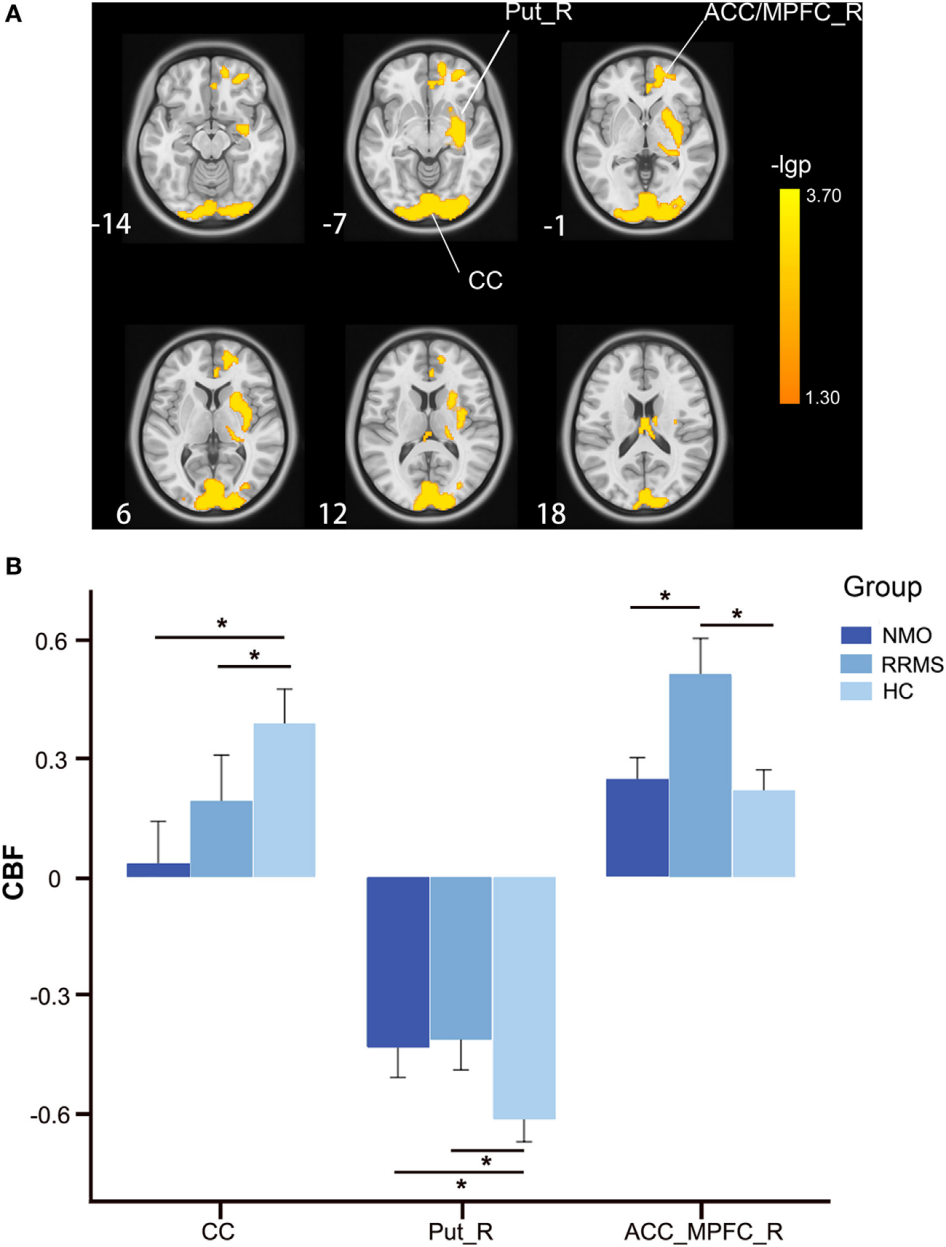
CBF differences among the relapsing-remitting multiple sclerosis, NMO, and control groups after GMV correction (*P* < 0.05, FWE-corrected). **(A)** Voxel-based analysis shows brain regions with significant CBF differences among the three groups using age and gender as nuisance variables (*P* < 0.05, FWE-corrected). **(B)** Bar plots show CBF differences between every two groups. Error bars are SEs. *P < 0.05. Abbreviations: ACC, anterior cingulate cortex; CBF, cerebral blood flow; CC, calcarine cortex; FWE, family-wise error; L, left; MPFC, medial prefrontal cortex; Put, putamen; R, right; GMV, gray matter volume.

By adding the GMV of each voxel as an additional covariate of no interest, we repeated the voxel-wise CBF comparisons and found significant CBF differences among the three groups in the bilateral CC and the right putamen and ACC/MPFC (*P* < 0.05, FWE correction) (Figure 2A; Table 2). The bilateral thalamus, the right sgACC/OG, and the left putamen and STG no longer showed CBF differences among the three groups after GMV correction.

The *post hoc* analyses found that both the RRMS and NMO patients showed increased CBF in the right putamen (*P* < 0.001 for both RRMS and NMO), and decreased CBF in the bilateral CC (*P* = 0.016 for RRMS and *P* < 0.001 for NMO) compared to the HC (Figure 2B). The RRMS patients showed increased CBF in the right ACC/MPFC compared to the NMO patients (*P* = 0.002) and HC (*P* < 0.001) (Figure 2B).

### The GMV Differences Among the Three Groups

After controlling for the effects of age and gender, the bilateral thalamus, the left inferior temporal gyrus, fusiform gyrus, ACC and caudate nucleus, and the right CC, gyrus rectus and putamen showed significant GMV differences (*P* < 0.01, FWE-corrected, threshold of cluster size = 100) among the three groups. The *post hoc* analyses revealed that both the RRMS and NMO patients showed GMV reductions in the bilateral thalamus, the left inferior temporal gyrus, fusiform gyrus, ACC and caudate nucleus, and the right gyrus rectus and putamen (*P* < 0.05 for both RRMS and NMO). The NMO patients additionally showed decreased GMV in the right CC compared to the HC (*P* < 0.05). The details are shown in Supplementary Material.

### Correlations of CBF Changes With Clinical Parameters

**Figure 3:**
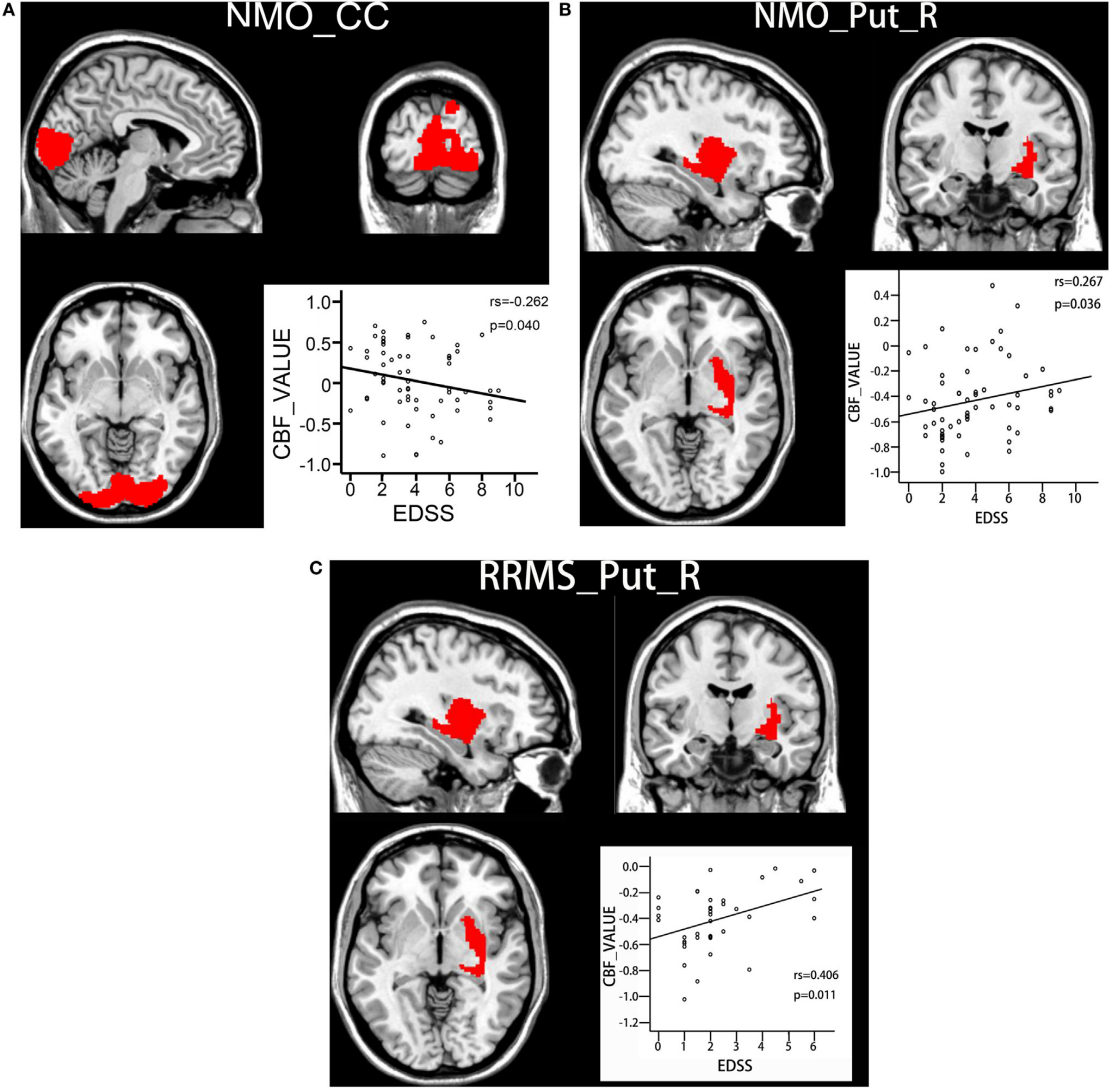
Correlations between CBF values and clinical disability in both patient groups. **(A)** Spearman correlations between CBF values in the bilateral calcarine cortex and EDSS scores in NMO patients. **(B)** Spearman correlations between CBF values in the right putamen and EDSS scores in NMO patients. **(C)** Spearman correlations between CBF values in the right putamen and EDSS scores in RRMS patients. Abbreviations: CBF, cerebral blood flow; CC, calcarine cortex; EDSS: Expanded Disability Status Scale; RRMS, relapsing-remitting multiple sclerosis; NMO, neuromyelitis optica; Put, putamen; R, right.

Spearman correlation analyses were used to investigate the correlations between CBF values in the brain regions with significant intergroup differences and EDSS scores. For the brain regions with reduced CBF in the NMO patients, there was a significant negative correlation (rs = −0.262, *P* = 0.04) between CBF in the bilateral CC and EDSS scores in the NMO patients (Figure 3A). For the brain regions with increased CBF in both the NMO and MS patients, there were significant positive correlations between CBF in the right putamen and EDSS scores in both the NMO (rs = 0.267, *P* = 0.036) (Figure 3B) and RRMS (rs = 0.406, *P* = 0.011) patients (Figure 3C). Moreover, voxel-wise correlation analyses were carried out to explore correlations between gray matter CBF values and EDSS scores in each patient group after controlling for age and gender (*P* < 0.001, uncorrected). We found that most of the brain regions showed positive or negative correlations between CBF values and EDSS scores in both patient groups. The distribution of the brain regions with significant correlations was different between NMO and MS patients. The details are shown in Supplementary Material.

## Discussion

In this study, we investigated the CBF changes in RRMS and NMO with and without GMV correction. Significant intergroup CBF differences in several brain regions diminished after GMV correction, suggesting that the CBF differences in these regions are possibly related to GMV changes. Three brain regions showed between-group CBF differences that were independent of the GMV changes. Two of these brain regions had similar CBF changes in the RRMS and NMO patients; however, the CBF in the medial prefrontal cortex (MPFC) differed in RRMS from that in NMO, indicating this feature as a potential imaging measure that may be used to distinguish RRMS from NMO. In addition, the CBF changes in the CC and the right putamen were correlated with clinical parameters in the corresponding patient groups.

### The Possible Mechanisms for CBF Changes in RRMS and NMO

Several mechanisms may account for CBF abnormality in RRMS and NMO. First, any factors impairing cerebral blood vessels would lead to CBF abnormalities. Perivascular inflammation is one of the prominent pathological features in both RRMS and NMO (22) that may impair the microvasculature and affect the precise regulation of CBF (21, 22, 36–39). Second, the integrity of the neurovascular unit (neurons, astrocytes, and vessels) is critically important for normal neurovascular coupling (40, 41). From the perspective of the normal neurovascular coupling, neuronal loss or dysfunction may reduce the metabolic demand and result in the CBF reduction (42–44), which may explain why CBF differences in several brain regions disappeared after GMV correction. In the neurovascular unit, astrocytes play an important role in bridging neuronal activity and vascular response (40, 41, 45). The foot processes of astrocytes are the main target of the NMO-IgG (21, 46–48), which leads to astrocyte dysfunction and impairments in the normal neurovascular coupling in NMO (42–44). Additionally, any factors influencing the signal transmission from neurons to astrocytes or from astrocytes to vascular structures may affect normal neurovascular coupling and lead to CBF changes. Cortical neuronal loss and reduced cortical perfusion due to anterograde or retrograde degeneration of axons should also be considered (36, 49–51), especially trans-synaptic degeneration (52–56). Finally, other factors such as neurotransmitters and non-specific agents [such as ET-1 transcription (57) and calcium increase (37)] that can affect vascular response may also contribute to CBF changes.

### CBF Differences Dependent on GMV Alterations

We found that significant CBF differences in several brain regions disappeared after GMV correction, including the bilateral thalamus, the left putamen and STG, and the right subgenual ACC and olfactory gyrus, most of which have shown GMV alterations in RRMS and/or NMO patients both in our study and in others (10, 12, 36, 58–63). This was also mainly verified by Spearman correlation analyses between the CBF values and GMV values in the patient groups (Supplementary Material). The GMV damage in these regions may be caused by neuronal degeneration within the cortex and/or may be secondary to the damage in other structures such as the brain white matter, spinal cord, and optic nerves (36). In these regions, the altered CBF could be better explained by the relatively normal neurovascular coupling. For example, the reduced number of neurons in regions with GMV reductions may reduce the metabolic demand and then reduce CBF. Therefore, we believe that the disappearance of significant CBF differences in several brain regions after GMV correction indicates that the altered CBF in these regions is possibly caused by GMV changes. However, the number of optic neuritis attacks may be a potential confounder, so limited reproducibility across VBM studies has been shown in regard to NMO (16). It should be noted that the relationship between CBF and GMV is rather complex and needs to be further investigated.

### CBF Differences Independent of GMV Alterations

The CC showed CBF reductions in both RRMS and NMO patients, which was consistent with prior findings in these two disorders (64–66). One possible explanation for the CBF change is the GMV reduction, which has been frequently reported in RRMS and NMO. However, the GMV reduction cannot fully explain the CBF reduction because we still found CBF reductions after GMV correction. Damage to the visual pathway may be an important reason for the dysfunction in the visual cortex, such as trans-synaptic degeneration of the anterior part of the visual pathway (52–56); in addition, the number of optic neuritis attacks in relapsing NMO has been claimed to be related to perfusion changes and clinical disabilities (13). The impairments in other components of this cortex may also be related to the CBF reductions, such as the selective immunological damage to oligodendrocytes in RRMS and to astrocytes in NMO and the damage to vascular structures due to the inflammation in both disorders. We also found that greater CBF reductions in this region resulted in more severe clinical disabilities in the NMO patients (both cluster-based and voxel-wise correlation analyses), which may be used as an imaging marker to objectively assess clinical disability in NMO.

We also found increased CBF in the right putamen in both the NMO and RRMS patients. The putamen, a part of the dorsal striatum that is involved in cognition and movement regulation and coordination, has been demonstrated to be impaired in the RRMS patients (12, 36, 58, 59). All factors affecting the neurovascular unit could theoretically result in abnormally increased CBF in this region; however, the exact biological mechanisms should be clarified in future studies. More importantly, there was a significant positive correlation between the CBF values and EDSS scores in both of the patient groups (according to both the cluster-based and voxel-wise correlation analyses), suggesting that the abnormally increased CBF in this region may be related to the clinical disabilities in both disorders. These findings suggested that the increased CBF is either a reflection of functional abnormality or an indication of functional compensation for patients with more severe clinical disabilities.

The CBF of the right ACC/MPFC was increased in RRMS patients, but not in NMO patients. The ACC/MPFC is the core component of the default mode network (DMN), which is involved in introspection, episodic memory, and self-processing (67–69). The DMN coordinates with the executive control network to support goal-directed behaviors (68). Although we do not know the exact mechanism that is responsible for the CBF increase in this region in RRMS, we think it may be related to the cognitive impairment in RRMS patients, a notion that has been supported by the correlation between this region’s abnormalities and the cognitive decline in RRMS patients (9, 70). However, we did not find significant CBF differences in the ACC/MPFC between the NMO patients and HC. Moreover, the ACC/MPFC was the only region that showed significant CBF differences between the RRMS and NMO patients, implicating it as a potential imaging marker to distinguish RRMS from NMO, but further validation is needed. The distinct CBF changes in RRMS and NMO may be related to the differences in neuropathological, immunologic, and other features between the two disorders. For example, cortical demyelinating lesions are frequently present in RRMS but are not detected in NMO (4).

### Limitations

This was a cross-sectional study; thus, we could not investigate the longitudinal changes in gray matter CBF. We did not exclude the effect of gray matter lesions because we consider these lesions as a feature of these demyelination disorders, which may have led to an overestimation of the CBF in some brain regions. In addition, we performed GMV correction to find alterations in perfusion independent of GM atrophy; this may not have eliminated the possible effects of GM volume differences at all, and a longitudinal study is a better choice to reveal the exact relationship between the CBF and GMV changes. Regarding CBF difference in the MPFC between RRMS and NMO, it is clear that further validation is definitely needed. While the RRMS patients showed reduced CBF in the CC, whether this finding suggested a high prevalence of optic neuritis in MS patients also requires further study. We did not evaluate the CBF changes in the white matter, though they should be investigated in future studies because functional and structural changes in the white matter are the key to a better understanding of RRMS and NMO. According to the proportion (40/62) of seropositive for AQP4 antibody, the NMO cohort may be heterogeneous and may have comprised patients with seropositive for myelin oligodendrocyte glycoprotein (MOG) antibody (71). Most of patients with RRMS and NMO had received immunological treatments that might have affected the CBF changes. Further work with drug-free patients will be necessary to confirm our results.

## Conclusion

We used a 3D pcASL technique to compare CBF differences among the RRMS, NMO, and control groups. Some brain regions demonstrated CBF changes related to GMV alterations, but others demonstrated CBF differences independent of GMV changes, and some regional CBF changes were correlated with the clinical disabilities. More importantly, we found CBF differences in the MPFC between the RRMS and NMO patients, which may be helpful in distinguishing RRMS from NMO after further validation.

## Ethics Statement

This study was carried out in accordance with the recommendations of “Medical Research Ethics Committee at Tianjin Medical University General Hospital” with written informed consent from all subjects. All subjects gave written informed consent in accordance with the Declaration of Helsinki. The protocol was approved by the “Medical Research Ethics Committee at Tianjin Medical University General Hospital.”

## Author Contributions

XZ and CY designed research; XZ, XG, HC, NZ, JS, and QW performed research; YQ, LZ, LY, and F-DS was involved in the clinical assessment; XZ, XG, and CY analyzed data; XZ, XG, and CY wrote the paper.

## Conflict of Interest Statement

The authors declare that the research was conducted in the absence of any commercial or financial relationships that could be construed as a potential conflict of interest.

## Footnotes

^1^http://www.fil.ion.ucl.ac.uk/spm/software/spm8/ (Accessed: March 02, 2015).

^2^http://www.fmrib.ox.ac.uk/fsl (Accessed: March 02, 2012).
