# Supplementary material for: Cerebral Blood Flow Changes in Multiple Sclerosis and Neuromyelitis Optica and Their Correlations With Clinical Disability
Source: Front Neurol. 2018 May 2;9:305. doi: 10.3389/fneur.2018.00305 (PMC5946009; doi:10.3389/fneur.2018.00305)
Supplement: Supplementary file 1 [file presentation_1.PDF]

Supplementary Material

Cerebral blood flow changes in multiple sclerosis and neuromyelitis optica and their correlations with clinical disability

Xue Zhang, MSc *, Xi Guo, MSc; Ningnannan Zhang, MD; Huanhuan Cai, MSc; Jie Sun, MSc; Qiuhui Wang, MD; Yuan Qi, MSc; Linjie Zhang, MD; Li Yang, MD; Fu-Dong Shi, MD

*** Correspondence:** Chunshui Yu, MD; chunshuiyu@tijmu.edu.cn.

1. **The inclusion criteria for NMO patients fulfilled the revised Wingerchuk diagnostic criteria** (1) **(Table S1), and a retrospective evaluation confirmed that they also satisfied the 2015 criteria for NMO spectrum disorder (NMOSD)** (2) **(Table S2).**

Table S1. Detailed information of NMO diagnosis according to the 2006 NMO Wingerchuk criteria

	2006 NMO Wingerchuk criteria				
	Main Criteria		Supportive Criteria		
Subject	Optic neuritis	Acute myelitis	Contiguous spinal cord MRI lesion extending over 3 vertebral segments	Brain MRI not meeting diagnostic criteria for multiple sclerosis	NMO-IgG seropositive status
No.001	√	√	√	×	AQP4+
No.002	√	√	×	√	AQP4+
No.003	√	√	√	√	AQP4+
No.004	√	√	√	√	AQP4-
No.005	√	√	×	√	AQP4+

No.006	√	√	√	√	AQP4-
No.007	√	√	√	×	AQP4+
No.008	√	√	√	√	AQP4-
No.009	√	√	×	√	AQP4+
No.010	√	√	√	√	AQP4+
No.011	√	√	√	√	AQP4+
No.012	√	√	√	×	AQP4+
No.013	√	√	×	√	AQP4+
No.014	√	√	√	√	AQP4-
No.015	√	√	×	√	AQP4+
No.016	√	√	√	√	AQP4-
No.017	√	√	√	√	AQP4-
No.018	√	√	×	√	AQP4+
No.019	√	√	×	√	AQP4+
No.020	√	√	√	√	AQP4+
No.021	√	√	√	√	AQP4+
No.022	√	√	√	√	AQP4+
No.023	√	√	×	√	AQP4+
No.024	√	√	√	√	AQP4+
No.025	√	√	√	√	AQP4-
No.026	√	√	√	√	AQP4-
No.027	√	√	√	√	AQP4+
No.028	√	√	√	√	AQP4-

No.029	√	√	×	√	AQP4+
No.030	√	√	√	√	AQP4+
No.031	√	√	√	×	AQP4+
No.032	√	√	√	√	AQP4+
No.033	√	√	√	√	AQP4+
No.034	√	√	√	√	AQP4-
No.035	√	√	×	√	AQP4+
No.036	√	√	√	√	AQP4-
No.037	√	√	√	√	AQP4+
No.038	√	√	√	√	AQP4+
No.039	√	√	√	√	AQP4-
No.040	√	√	×	√	AQP4+
No.041	√	√	√	√	AQP4-
No.042	√	√	√	√	AQP4+
No.043	√	√	√	√	AQP4-
No.044	√	√	√	×	AQP4+
No.045	√	√	√	√	AQP4+
No.046	√	√	√	√	AQP4-
No.047	√	√	√	√	AQP4+
No.048	√	√	√	√	AQP4+
No.049	√	√	×	√	AQP4+
No.050	√	√	√	√	AQP4+
No.051	√	√	√	√	AQP4+
No.052	√	√	√	√	AQP4+

No.053	√	√	√	√	AQP4-
No.054	√	√	√	√	AQP4-
No.055	√	√	√	√	AQP4-
No.056	√	√	√	√	AQP4-
No.057	√	√	×	√	AQP4+
No.058	√	√	√	√	AQP4-
No.059	√	√	√	√	AQP4-
No.060	√	√	√	√	AQP4+
No.061	√	√	√	√	AQP4+
No.062	√	√	√	√	AQP4-

AQP4: aquaporin-4; IgG: immunoglobulin G; MRI: magnetic resonance imaging; NMO, neuromyelitis optica.

√: conformity; ×: non- conformity.

Table S2. Detailed information of NMO diagnosis according to the 2015 International consensus diagnostic criteria for NMOSD

2015 International consensus diagnostic criteria for neuromyelitis optica spectrum disorders							
Subject	NMO-IgG seropositive status	Optic neuritis	Acute myelitis	Area postrema syndrome	Acute brainstem syndrome	Symptomatic narcolepsy or acute diencephalic clinical syndrome with NMOSD-typical diencephalic MRI lesions	Symptomatic cerebral syndrome with NMOSD-typical brain lesions
No.001	AQP4+	√	√	×	×	√	×
No.002	AQP4+	√	√	×	×	×	×
No.003	AQP4+	√	√	×	√	×	√
No.004	AQP4-	√	√	√	×	×	√
No.005	AQP4+	√	√	×	×	×	×
No.006	AQP4-	√	√	×	×	×	×
No.007	AQP4+	√	√	×	×	×	×
No.008	AQP4-	√	√	×	×	×	×
No.009	AQP4+	√	√	×	×	×	×

No.010	AQP4+	√	√	√	×	×	×
No.011	AQP4+	√	√	×	×	×	×
No.012	AQP4+	√	√	×	×	×	√
No.013	AQP4+	√	√	×	×	×	×
No.014	AQP4-	√	√	×	×	×	√
No.015	AQP4+	√	√	×	×	×	√
No.016	AQP4-	√	√	√	×	√	×
No.017	AQP4-	√	√	×	×	×	×
No.018	AQP4+	√	√	×	×	×	√
No.019	AQP4+	√	√	×	×	×	×
No.020	AQP4+	√	√	×	×	×	×
No.021	AQP4+	√	√	×	×	×	×
No.022	AQP4+	√	√	×	×	×	×
No.023	AQP4+	√	√	×	×	×	×
No.024	AQP4+	√	√	×	×	×	√
No.025	AQP4-	√	√	×	×	×	√
No.026	AQP4-	√	√	×	×	×	√

No.027	AQP4+	√	√	×	×	×	√
No.028	AQP4-	√	√	×	×	×	×
No.029	AQP4+	√	√	×	×	×	×
No.030	AQP4+	√	√	×	×	×	×
No.031	AQP4+	√	√	×	×	×	×
No.032	AQP4+	√	√	×	×	×	×
No.033	AQP4+	√	√	×	×	×	×
No.034	AQP4-	√	√	×	×	×	√
No.035	AQP4+	√	√	×	×	×	×
No.036	AQP4-	√	√	×	×	×	×
No.037	AQP4+	√	√	×	×	×	√
No.038	AQP4+	√	√	×	×	×	√
No.039	AQP4-	√	√	×	×	×	×
No.040	AQP4+	√	√	√	×	×	√
No.041	AQP4-	√	√	×	×	×	×
No.042	AQP4+	√	√	×	×	×	×
No.043	AQP4-	√	√	×	×	×	×

No.044	AQP4+	√	√	×	×	×	√
No.045	AQP4+	√	√	×	×	×	√
No.046	AQP4-	√	√	×	×	×	×
No.047	AQP4+	√	√	×	×	×	√
No.048	AQP4+	√	√	×	×	×	√
No.049	AQP4+	√	√	×	×	×	×
No.050	AQP4+	√	√	×	×	×	√
No.051	AQP4+	√	√	×	×	×	√
No.052	AQP4+	√	√	×	×	×	×
No.053	AQP4-	√	√	×	×	×	×
No.054	AQP4-	√	√	×	×	×	×
No.055	AQP4-	√	√	×	×	×	√
No.056	AQP4-	√	√	×	×	×	√
No.057	AQP4+	√	√	×	×	×	×
No.058	AQP4-	√	√	×	×	×	√
No.059	AQP4-	√	√	×	×	×	√
No.060	AQP4+	√	√	√	×	×	√

No.061	AQP4+	√	√	×	×	×	√
No.062	AQP4-	√	√	×	×	×	×

AQP4: aquaporin-4; IgG: immunoglobulin G; MRI: magnetic resonance imaging; NMO, neuromyelitis optica; NMOSD, neuromyelitis optica spectrum disorders.

√: conformity; ×: non- conformity.

2. Correlations of CBF changes with GMV values

For clusters with significant CBF changes in RRMS or NMO groups, we performed Spearman correlations analysis between CBF values and GMV values in the patient group. There were significant positive correlations between CBF and GMV values in the right thalamus (rs = 0.407, P = 0.001 for NMO and rs = 0.597, P < 0.001 for RRMS), the right sgACC/OG (rs = 0.38, P = 0.002 for NMO and rs = 0.486, P = 0.002 for RRMS) and the left STG (rs = 0.311, P = 0.014 for NMO and rs = 0.668, P < 0.001 for RRMS), and significant negative correlations in bilateral putamen (left: rs = -0.384, P = 0.016; right: rs = -0.366, P = 0.022) in RRMS patients. These results could be evidences for explaining those clusters disappearance after GMV correction.

Supplementary Figure

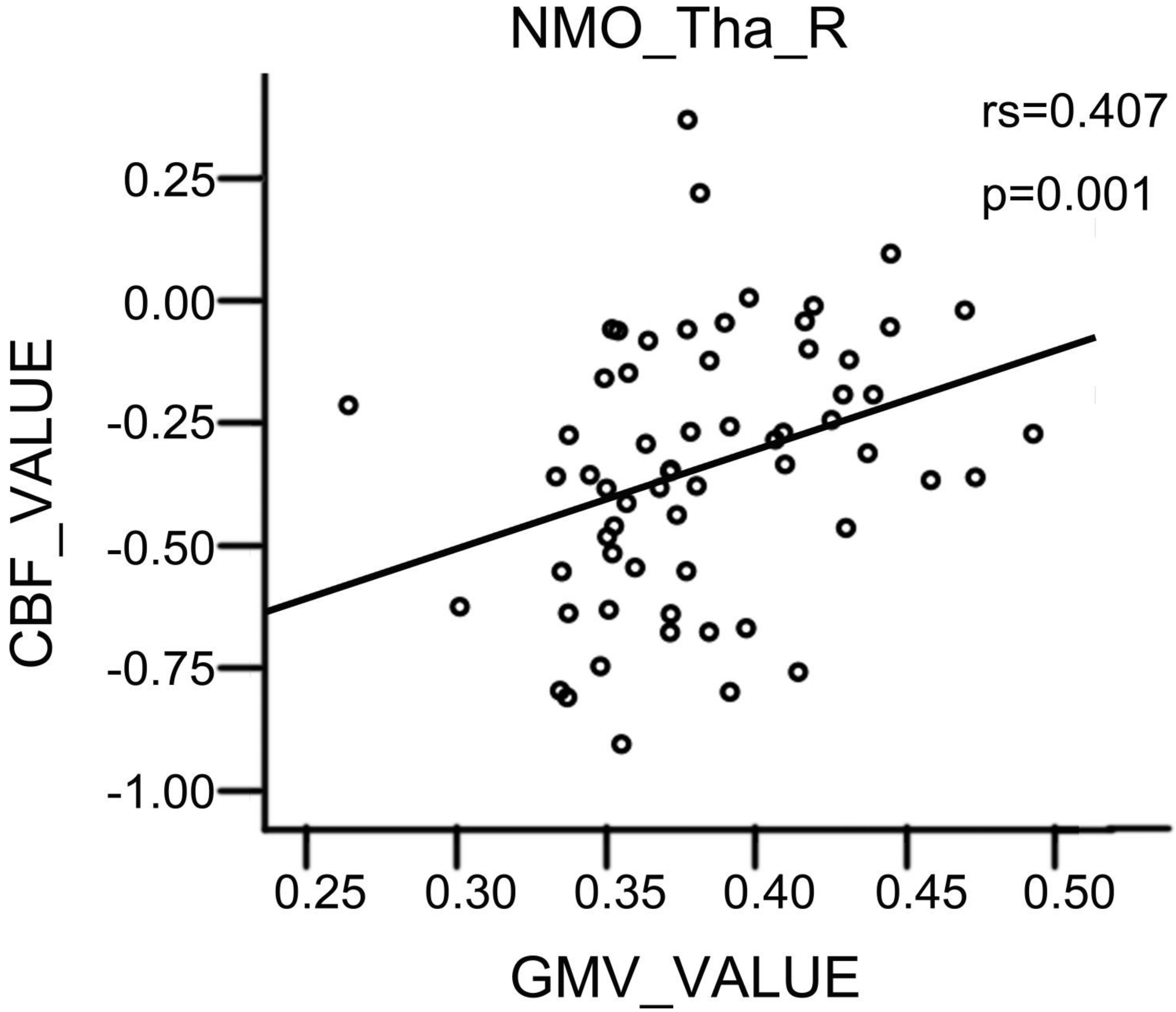

Supplementary Figure 1. A significant positive Spearman correlation between CBF and GMV values in the right thalamus in NMO patients (rs = 0.407, *P* = 0.001).

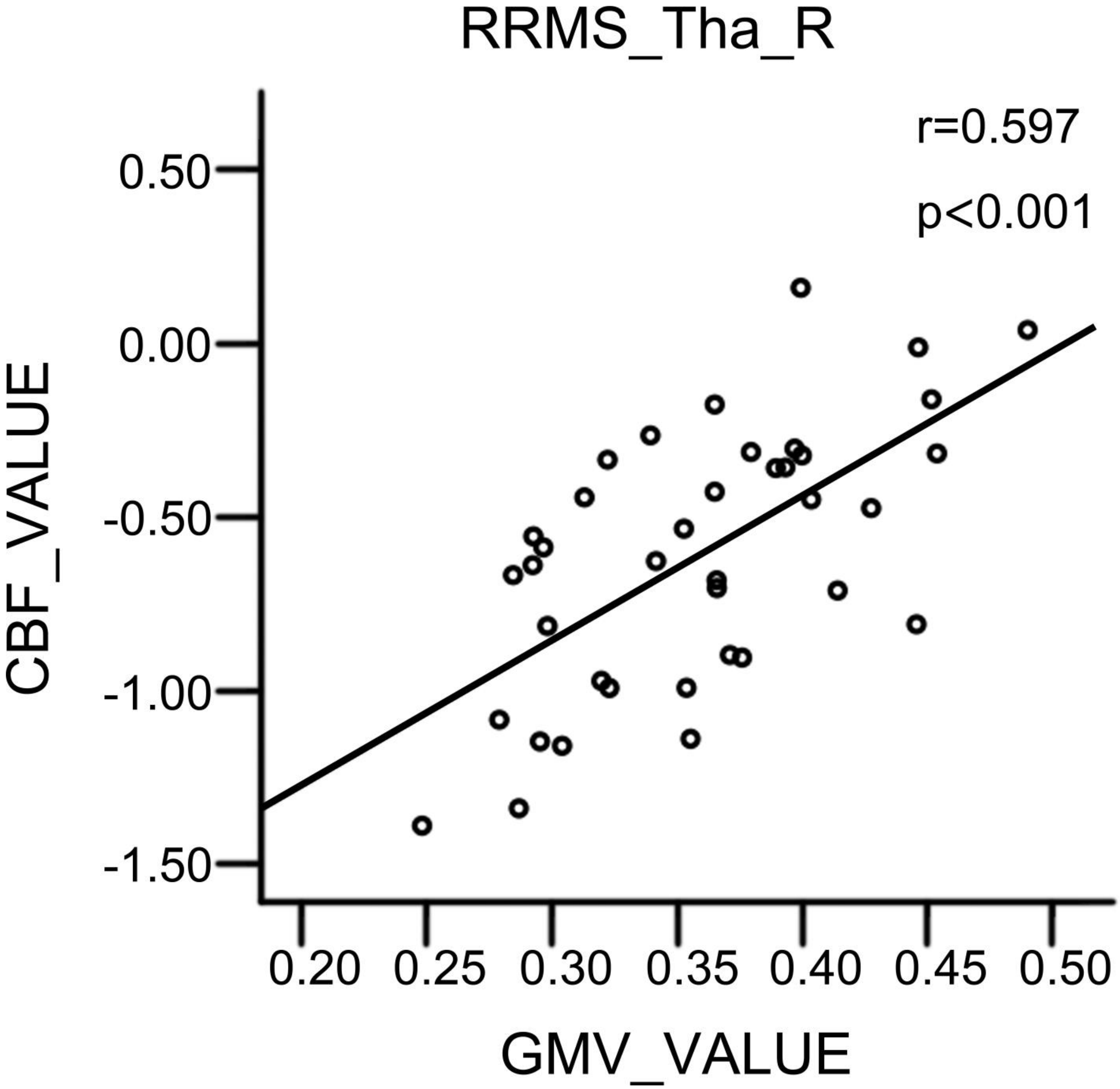

Supplementary Figure 2. A significant positive Spearman correlation between CBF and GMV values in the right thalamus in RRMS patients (rs = 0.597, $P < 0.001$).

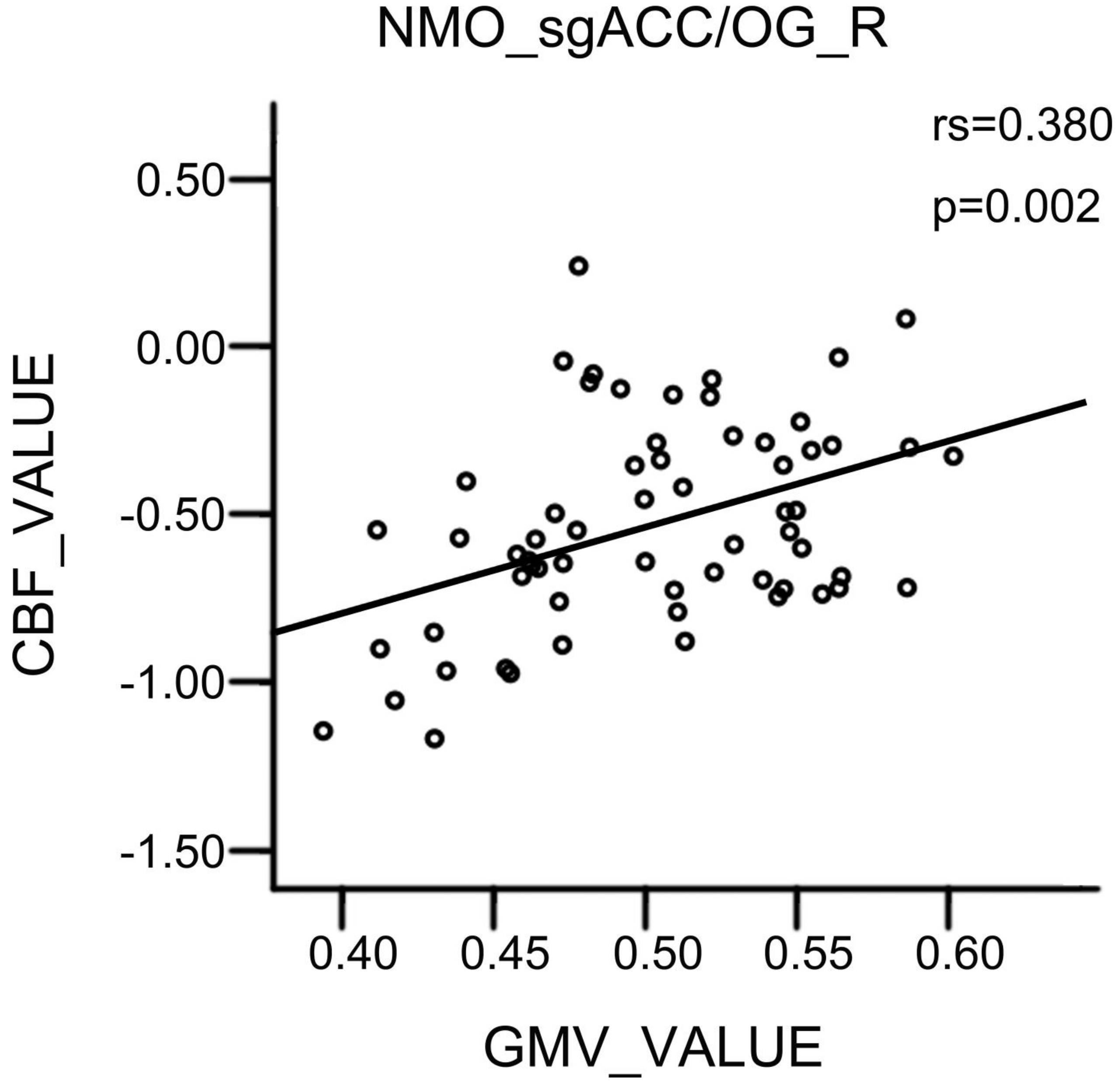

Supplementary Figure 3. A significant positive Spearman correlation between CBF and GMV values in the right sgACC/OG in NMO patients (rs = 0.380, $P = 0.002$).

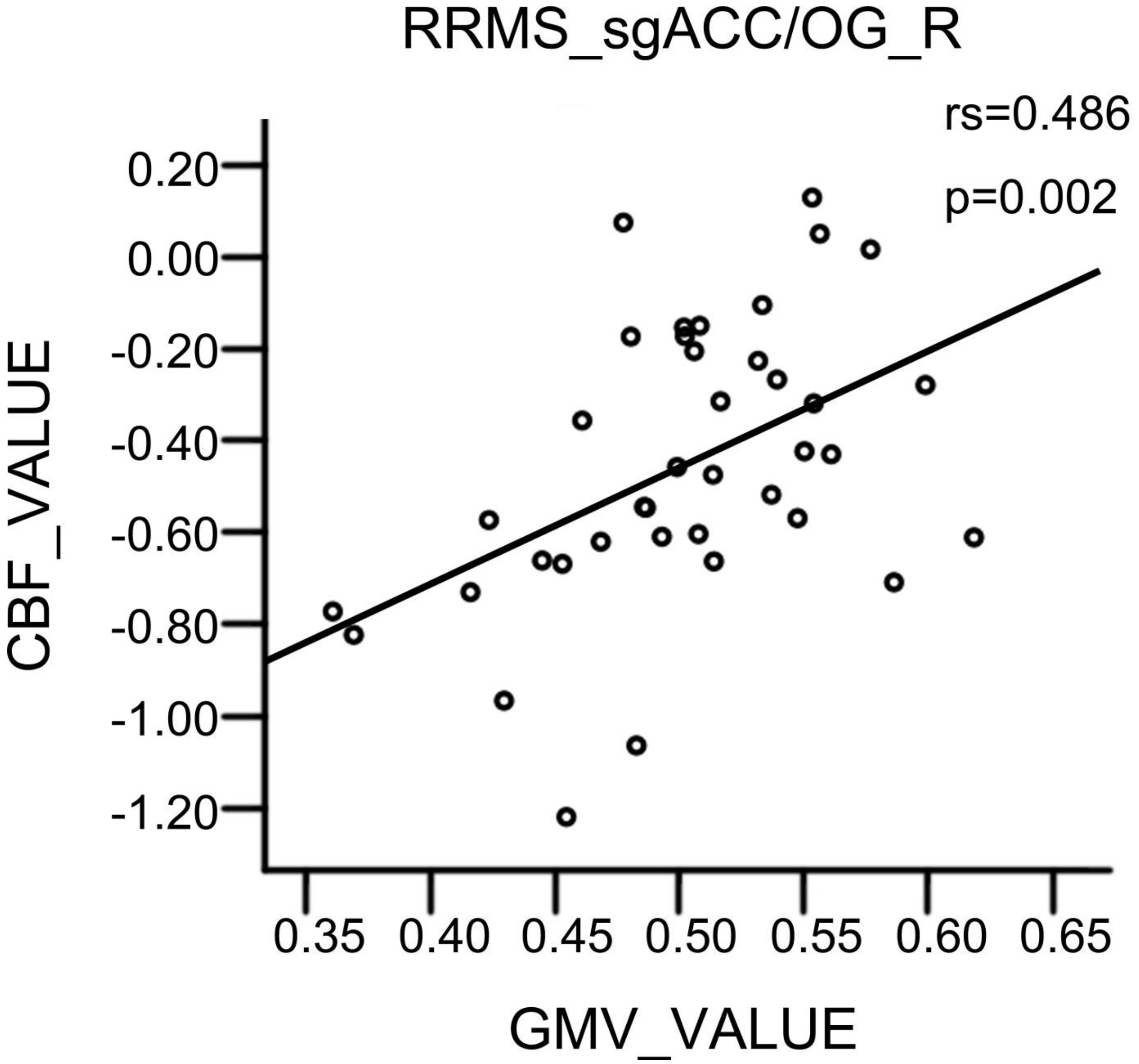

Supplementary Figure 4. A significant positive Spearman correlation between CBF and GMV values in the right sgACC/OG in RRMS patients (rs = 0.486, *P* = 0.002).

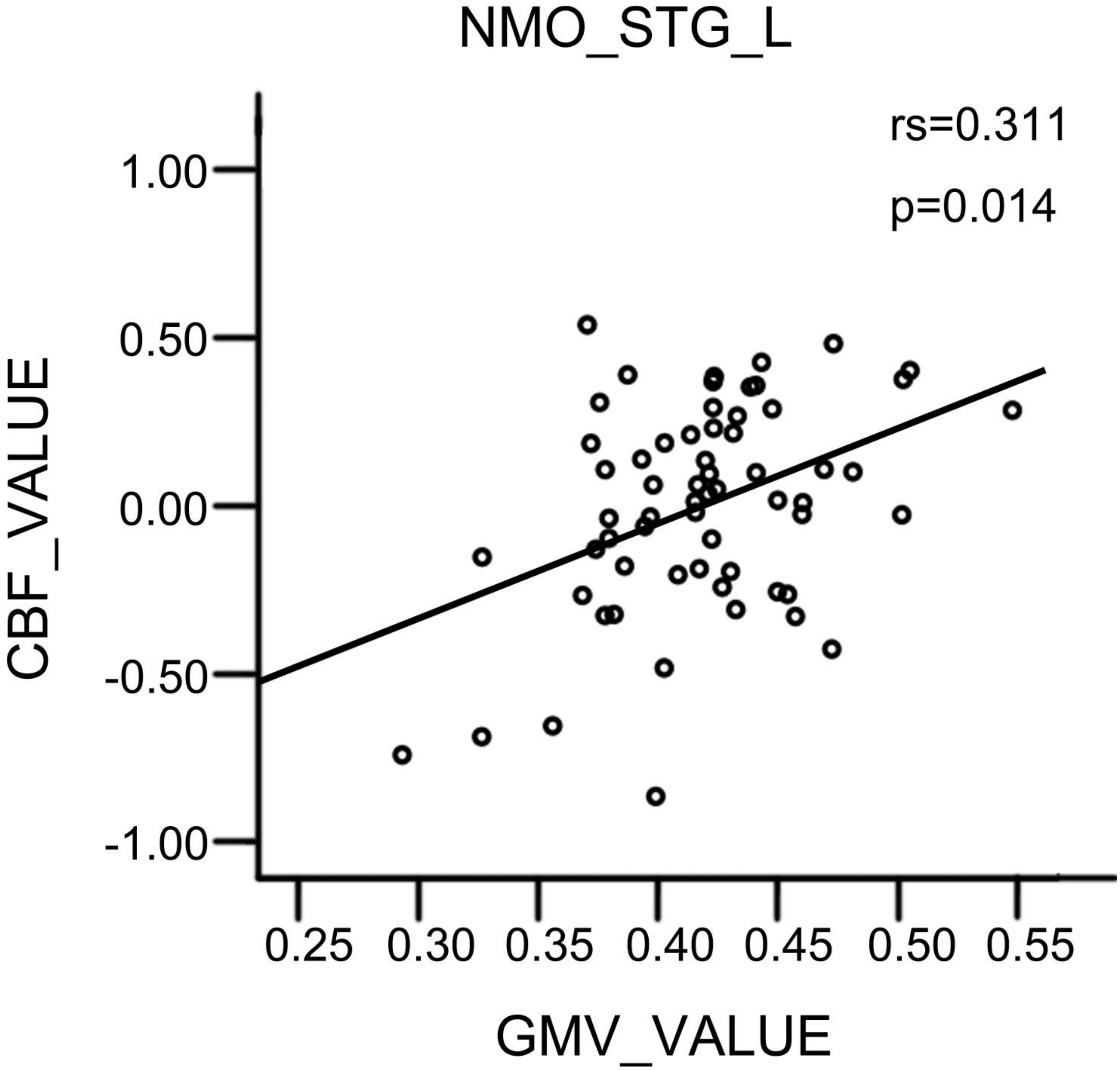

Supplementary Figure 5. A significant positive Spearman correlation between CBF and GMV values in the left STG in NMO patients (rs = 0.311, *P* = 0.014).

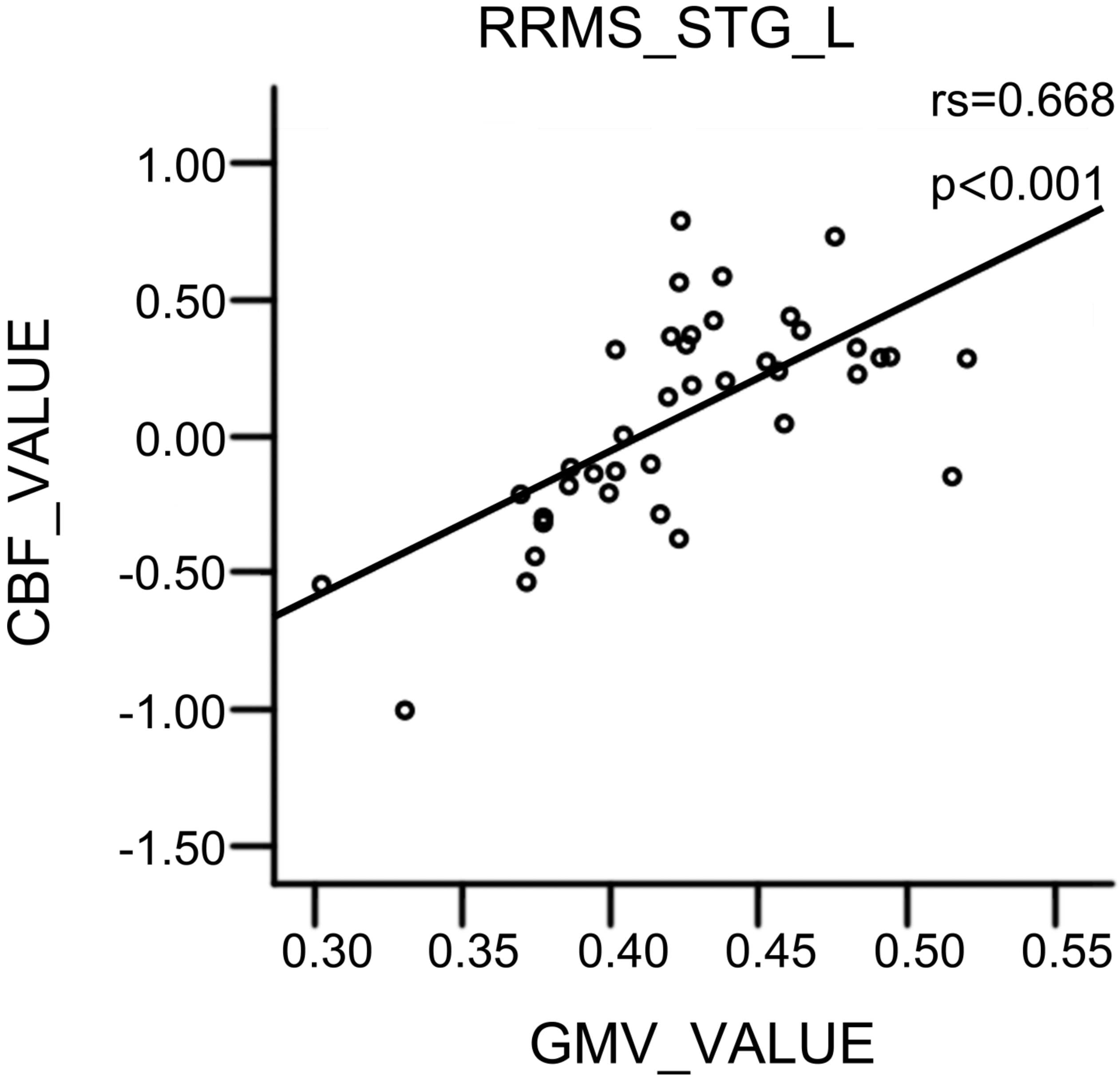

Supplementary Figure 6. A significant positive Spearman correlation between CBF and GMV values in the left STG in RRMS patients (rs = 0.668, $P < 0.001$).

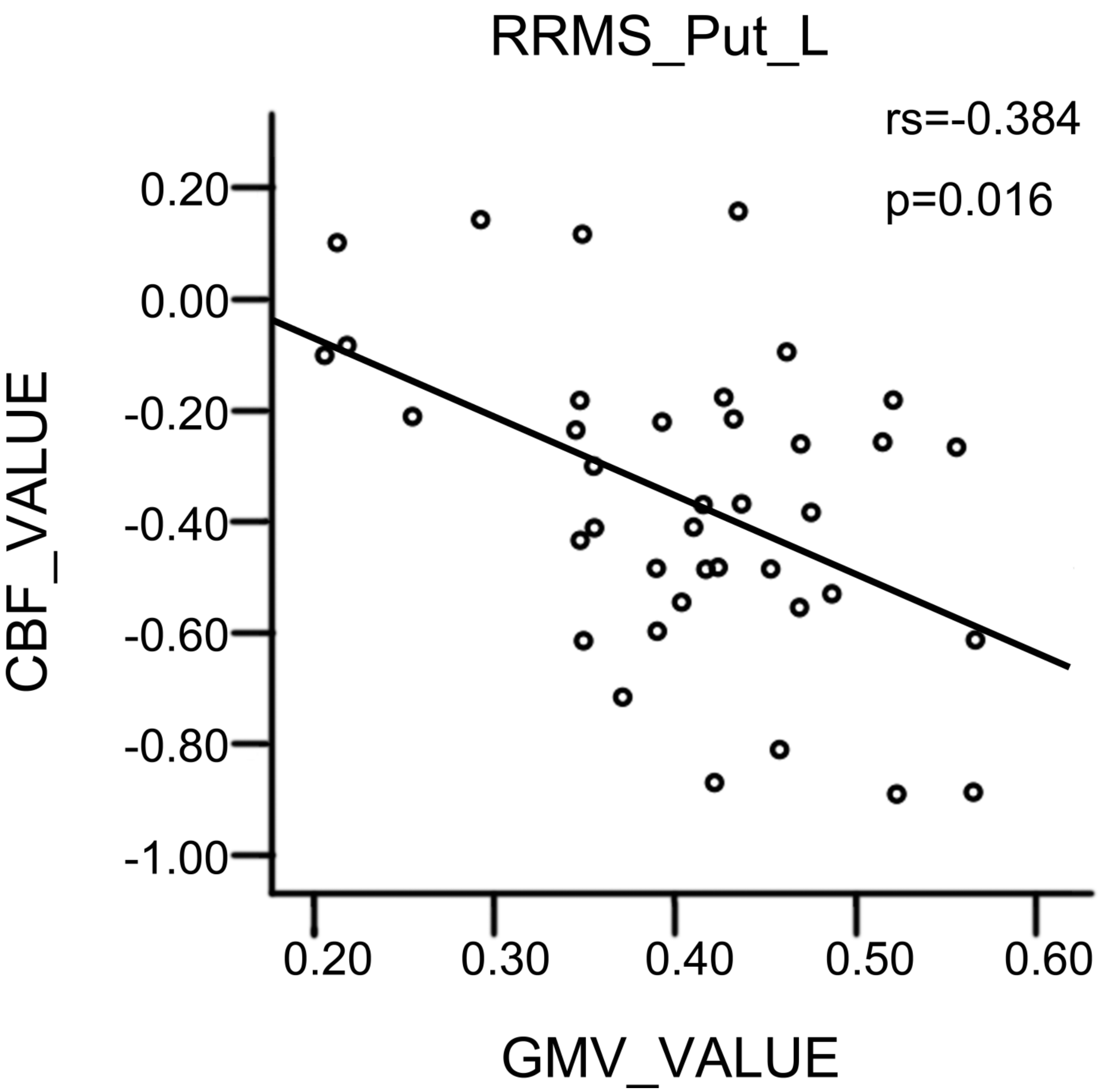

Supplementary Figure 7. A significant negative Spearman correlation between CBF and GMV values in the left putamen in RRMS patients (rs = -0.384, P = 0.016).

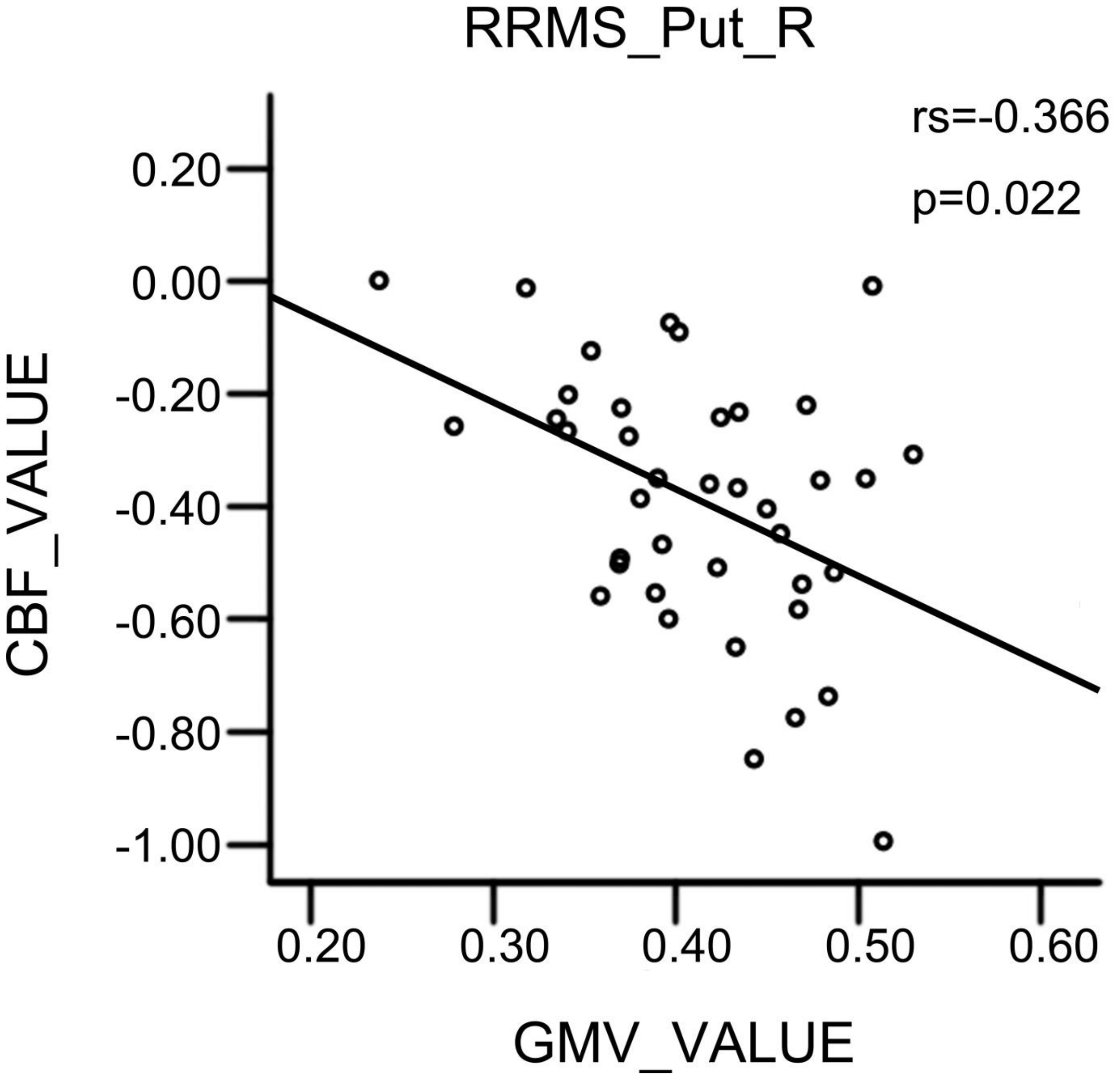

Supplementary Figure 8. A significant negative Spearman correlation between CBF and GMV values in the right putamen in RRMS patients (rs = -0.366, P = 0.022).

3. The GMV differences among the three groups

Non-parametric test was performed to investigate voxel-wise GMV differences among the RRMS, NMO, and HC groups, while controlling for the effects of age and gender. Multiple comparisons for these voxel-wise analyses were corrected using permutation-based non-parametric testing (Randomise v2.1 of FSL, http://www.fmrib.ox.ac.uk/fsl). The number of permutations was 5000 and the significance threshold for intergroup differences was set at $P < 0.01$ after correcting for family wise error (FWE) using the threshold-free cluster enhancement (TFCE) option in permutation-testing tool in FSL. For each subject, the GMV of each cluster with a significant group difference was extracted and used for region of interest (ROI)-based analyses. With age and sex as covariates of no interest, the Kruskal-Wallis ANCOVA was used to test GMV differences among the three groups and then *post-hoc* comparisons were used to identify GMV differences between every two groups ($P < 0.01$).

After controlling for the effects of age and gender, the bilateral thalamus, the left inferior temporal gyrus, fusiform gyrus, anterior cingulate cortex, caudate nucleus and the right calcarine cortex, gyrus rectus, and putamen showed significant GMV differences ($P < 0.01$, FWE-corrected, threshold of cluster size=100) among the three groups. The *post hoc* analyses revealed that both the RRMS and NMO patients showed GMV reductions in the bilateral thalamus and in the left inferior temporal gyrus, fusiform gyrus, anterior cingulate cortex, caudate nucleus and the right gyrus rectus, and putamen ($P < 0.05$ for both RRMS and NMO). The NMO patients additionally showed decreased GMV in the right calcarine cortex compared to the healthy controls ($P < 0.05$).

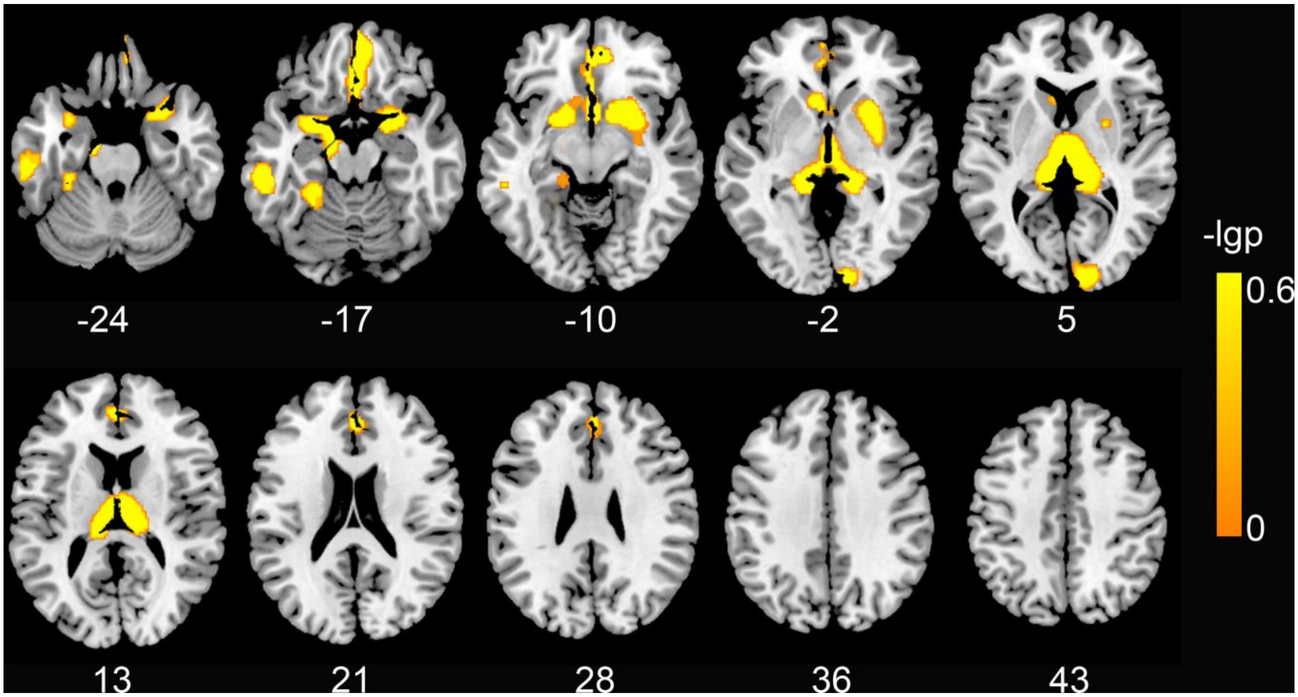

Supplementary Figure 9. GMV differences among the RRMS, NMO and control groups (P < 0.01, FWE corrected).

4. Voxel-wise correlation analysis between grey matter CBF changes and EDSS score

Voxel-wise multiple regression analysis was carried out to explore the correlation between the grey matter CBF changes and EDSS score across each group patients after controlling for the age and gender using permutation-based non-parametric testing (Randomise v2.1 of FSL, http://www.fmrib.ox.ac.uk/fsl). The permutation-based nonparametric inference was set as 5000 permutations. The correction for multiple correlations was processed using threshold-free cluster enhancement (TFCE) as it was generally more profound and avoided the necessity for the use of arbitrary cluster-forming threshold. The statistical threshold for significance was defined at $P < 0.001$.

Generally speaking, we found that positive correlations between grey matter CBF and EDSS scores mainly shown in the extensive frontal lobe, temporal lobe, partial parietal and limbic lobe and the bilateral putamen, thalamus; negative correlations mainly distributed in the temporal poles, occipital lobe (especially calcarine cortex) and partial frontal and parietal lobe in both MS and NMO patients, in addition, scale of negative correlations in NMO patients was more widespread than that in MS patients, such as the bilateral caudate nucleus, limbic and insula lobe.

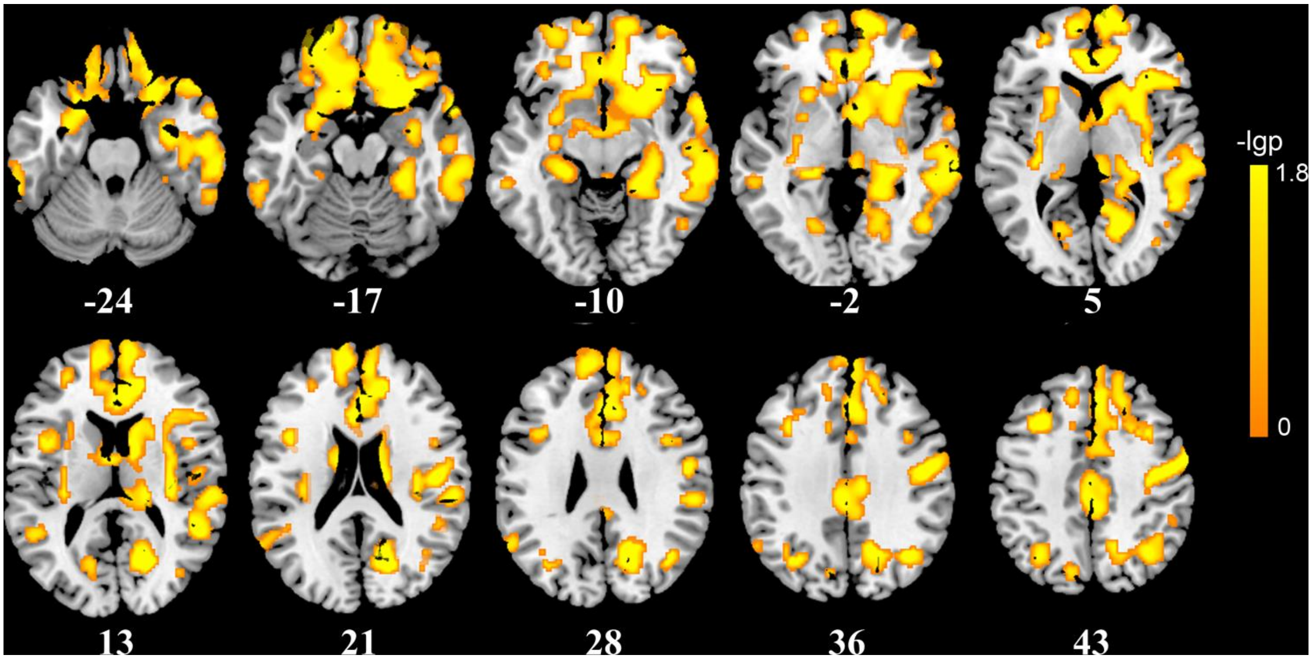

Supplementary Figure 10. Brain regions in which grey matter CBF showed positive correlation with EDSS scores on voxel-wise correlation analysis in MS patients after controlling for the age and gender ($P < 0.001$, threshold of cluster size =100).

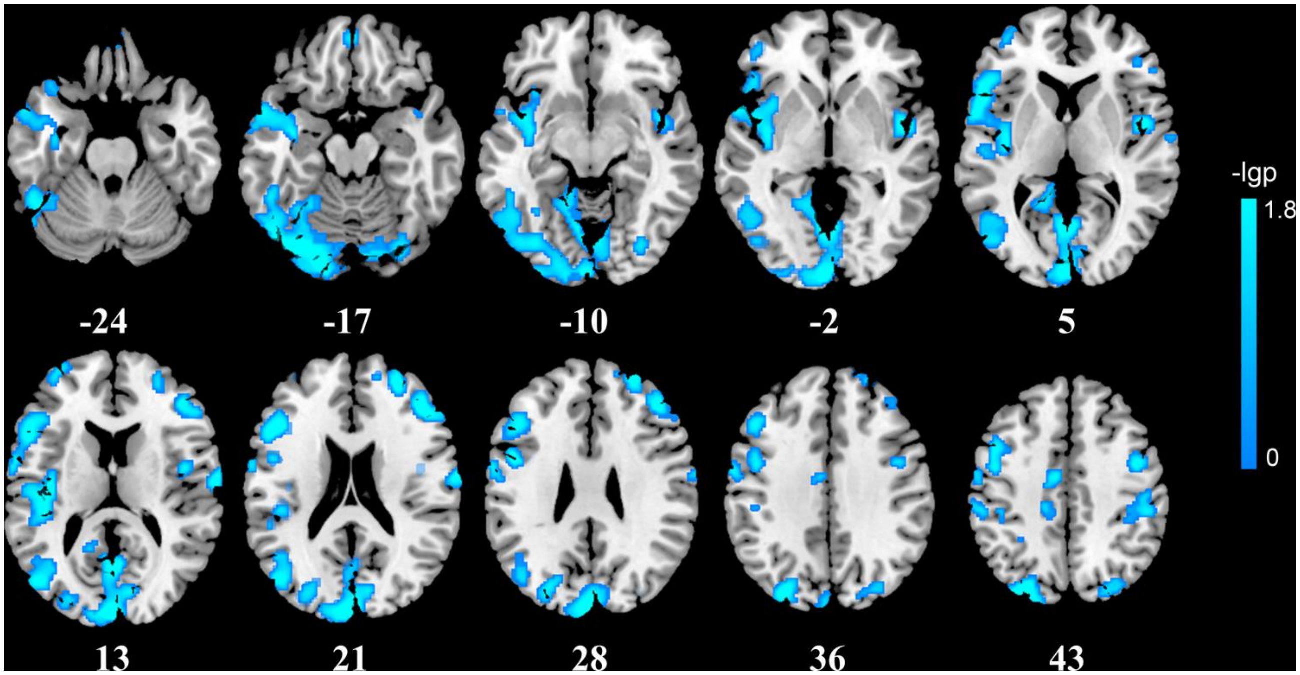

Supplementary Figure 11. Brain regions in which grey matter CBF showed negative correlation with EDSS scores on voxel-wise correlation analysis in MS patients after controlling for the age and gender (*P* < 0.001, threshold of cluster size =100).

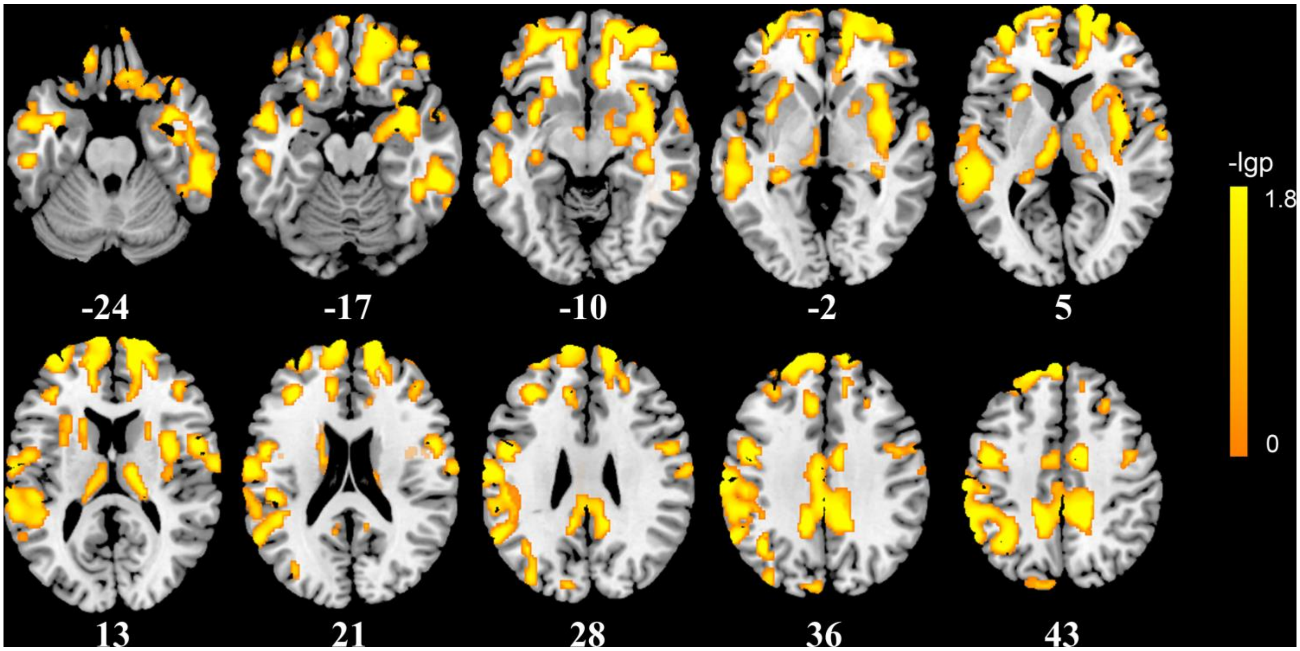

Supplementary Figure 12. Brain regions in which grey matter CBF showed positive correlation with EDSS scores on voxel-wise correlation analysis in NMO patients after controlling for the age and gender ($P < 0.001$, threshold of cluster size =100).

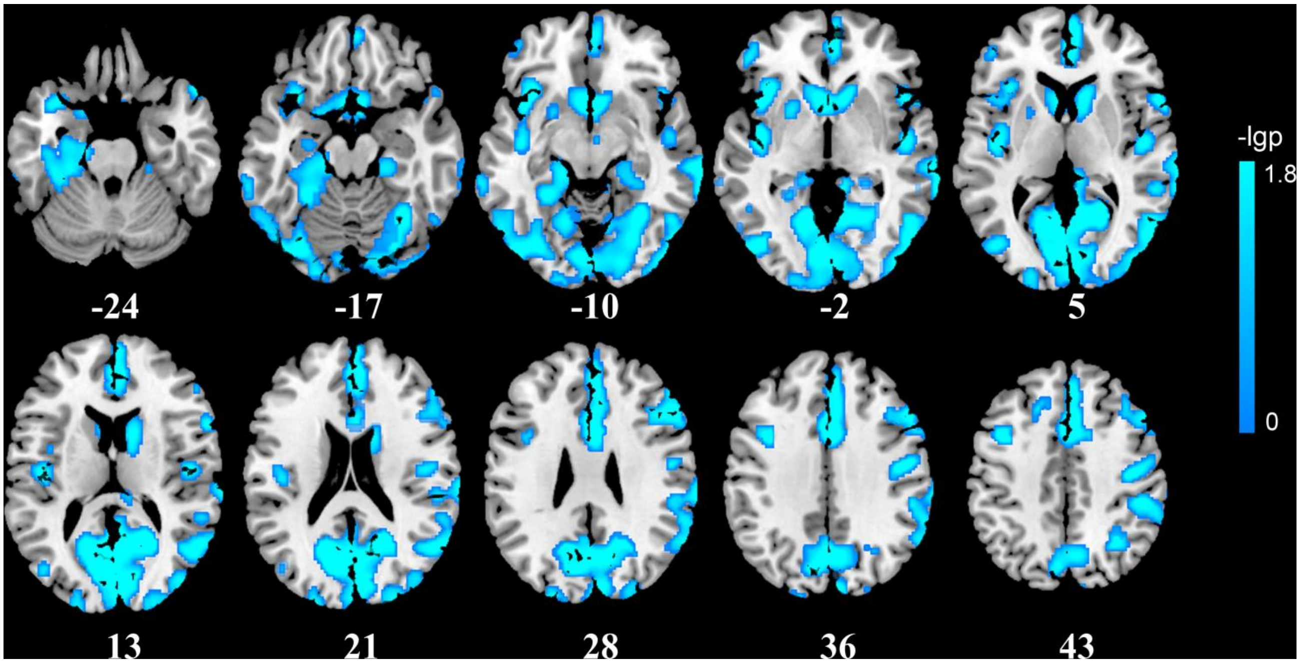

Supplementary Figure 13. Brain regions in which grey matter CBF showed negative correlation with EDSS scores on voxel-wise correlation analysis in NMO patients after controlling for the age and gender ($P < 0.001$, threshold of cluster size =100).

5. Detection of Antibodies against AQP4

AQP4-Ab was detected by the cell-based assay (CBA). 293 human embryonic kidney (HEK) cells were transfected with either enhanced green fluorescent protein (EGFP)-tagged human AQP4-M23-cDNA or EGFP alone, then incubated with the diluted serum. After washing, cells were fixed and then incubated with Alexa Fluor 568–conjugated antihuman IgG. Cells were washed and Olympus IX-71 fluorescence microscope was used to detect the binding of the sera to the cells. Values were scored on a scale of 0–4 independently by two individuals, values of one and above were considered positive (3). Test results of the AQP4 antibody in NMO patients were shown in Table S1 and Table S2.

Reference

1. Wingerchuk DM, Lennon VA, Pittock SJ, Lucchinetti CF, Weinshenker BG. Revised diagnostic criteria for neuromyelitis optica. *Neurology* (2006) **66**:1485–1489. doi:10.1212/01.wnl.0000216139.44259.74

2. Wingerchuk DM, Banwell B, Bennett JL, Cabre P, Carroll W, Chitnis T, de Seze J, Fujihara K, Greenberg B, Jacob A, et al. International consensus diagnostic criteria for neuromyelitis optica spectrum disorders. *Neurology* (2015) **85**:177–189. doi:10.1212/WNL.0000000000001729

3. Yang CS, Zhang DQ, Wang JH, Jin WN, Li MS, Liu J, Zhang CJ, Li T, Shi FD, Yang L. Clinical features and sera anti-aquaporin 4 antibody positivity in patients with demyelinating disorders of the central nervous system from Tianjin, China. *CNS Neurosci Ther* (2014) **20**:32–39. doi:10.1111/cns.12156
